# Hypothesizing Las Vegas and Sutherland Springs Mass Shooters Suffer from Reward Deficiency Syndrome: “Born Bad”

**DOI:** 10.17756/jrdsas.2017-038

**Published:** 2017-12-30

**Authors:** Thomas McLaughlin, Kenneth Blum, Bruce Steinberg, David Siwicki, Joseph Campione, Rajendra D. Badgaiyan, Eric R. Braverman, Edward J. Modestino, Marjorie C. Gondré—Lewis, David Baron, Deborah C. Mash, John Giordano, Panayotis K. Thanos

**Affiliations:** 1 Center for Psychiatric Medicine, North Andover, MA, USA; 2 Department of Psychiatry, University of Florida & McKnight Brain Institute, College of Florida, Gainesville, FL, USA; 3 Dominion Diagnostics, LLC, North Kingstown, RI, USA; 4 Department of Genetic Addiction Research, Geneus Health LLC, San Antonio, TX, USA; 5 Department of Psychology, Curry College, Milton, MA, USA; 6 Department of Psychiatry, Ichan School of Medicine, New York, NY, USA; 7 Department of Clinical Neurology, Path Foundation, New York, NY, USA; 8 Departments of Anatomy & Psychiatry, Howard University School of Medicine, Washington, DC, USA; 9 National Human Genome Center at Howard University, Washington, DC, USA; 10 Department of Psychiatry & Behavioral Sciences, Keck School of Medicine, University of Southern California, Los Angeles, CA, USA; 11 Department of Neurology, University of Miami Miller School of Medicine, Miami, FL, USA; 12 Division of Nutrigenomics and therapy, John Giordano’s Life Enhancement Aftercare Recovery Center, Ft. Lauderdale, FL, USA; 13 Behavioral Neuropharmacology and Neuroimaging Laboratory on Addictions, Research Institute on Addictions, University at Buffalo, Buffalo, NY, USA

**Keywords:** Reward Deficiency Syndrome (RDS), Hypodopaminergia, Guns, Mass shootings, Gambling, Hypersexuality, ADHD

## Abstract

The slaughters in Las Vegas and Sutherland Springs demand explanation, in the face of the ineffable. An understanding of the shooters’ motives could restore our trust in our mutually cooperative existence. In this short communication we provide post-hoc rationale of both Stephen Paddock (Las Vegas mass shooting) and Devin Kelley (Southerland Springs mass shooting) and hypothesize that these shooters had genetically induced “Reward Deficiency Syndrome” (RDS) and a hypodopaminergia trait/state. In this particular case we are in pursuit of trying to obtain postmortem samples of mass shooters for subsequent epigenetic and neurogenetic analyses. It is our contention that early genetic identification of RDS and its pathological behaviors including hyper – sexuality, violence, a love for guns, even in children, could be a giant step forward in potentially saving lives.

## Stephen Paddock

***“All he ever wanted was a freaken royal flush.”*** (Eric Paddock, brother)

News accounts indicate Stephen Paddock had an addiction to solitary gambling, an increasing fascination with guns, sexually sadistic, rape fantasies/acts, a tendency to be oppositional and to cheat without remorse, a need for benzodiazepine medication, and an alcohol use disorder (AUD).

What is known about his father, Patrick, is that he, too, was a gambler (avid bridge player), who used *machine guns* to rob banks and was regarded by the FBI as a “psychopath.” Although his father was incarcerated, when he was seven years old, Stephen would later state, “I didn’t have anything really to do with him, but the bad streak is in my blood. I was born bad.”

As Stephen aged, his range of interests narrowed as he became more obsessed with gambling and guns. A blog by Steven Kotler [1] links firing a firearm to activation of brain dopamine. This release could have caused an addiction to using an assault rifle, releasing as it does the main, brain reward chemical, dopamine, at the rate of 100 milliseconds every firing round.

With the exception of his final acts, there is little evidence to support a lifelong diagnosis of Antisocial Personality Disorder, Psychopathy or Pathological Aggression. Nevertheless, the homicidal, calculating nature of his crime demands consideration of these diagnoses, especially, given his father’s behavior and Stephen’s supposition that he, too, had his father’s traits.

Because of his heinous act, we posit the diagnoses of Antisocial Personality Disorder, in addition to Gambling Disorder, Socially Avoidant Personality Disorder, Sexual Sadism and Benzodiazepine Addiction to argue for a, primarily, genetic basis for his lifestyle and his motives [2]. (Reward Deficiency Syndrome (RDS), which we propose as an explanatory rubric, subsumes a variety of these and other diagnoses and is currently included as an abnormal psychological, genetically-based disorder in the SAGE Encyclopedia of Abnormal and Clinical Psychology) [3].

We hypothesize that Stephen Paddock had a genetically desensitized, brain reward circuit and, to keep overwhelming and malignant boredom at bay, as the sensitivity of his, critically important, dopamine D2 receptors faded with age (combined with his proposed endowment with low D2 receptors), nothing could alleviate his inner deadness, but the exciting prospect of a mass killing. As he had an escape plan, he anticipated notoriety, as, perhaps, another antidote to his boredom. (One gambling addict described his boredom, prior to a gambling episode as “*Like a deafening silence”*).

This hypothesis is supported by the absence of ideological fanaticism as well as any history of Pathological Aggression, Psychosis, Major Affective Illness (Major Depression or Bipolar Disorder) or Post-Traumatic-Stress Disorder.

More recent news accounts of the arrest of, his brother, Bruce Paddock, on child pornography charges also support the genetic basis of these behaviors, since “Hyper-Sexuality” as behavioral addiction, is also included under “Reward Deficiency Syndrome”.

### Reward Deficiency Syndrome Links (see Table 1)

Pastwa-Wojciechowska [4] noted that incarcerated men with the diagnosis of pathological gambling could be characterized by psychopathic personality disorders, alcohol problems and criminality. Moon et al. [5] reported Antisocial-Impulsive (AI) Gamblers have high rates of impulsivity and risk-taking. Moreover, Blaszczcynski et al. [6] found that pathological gamblers seek stimulation as a means of reducing aversive, under-aroused states of boredom. In addition, the fact that Paddock’s game of choice was “Video Poker,” which suggests a solitary loner quality, possibly, linked to Schizoid-Avoidant behavior, which has been associated with the A1 allele of the DRD2 gene and its concomitant, 30–40% reduction in D2 receptor number [7]. Without genetic information, the Unabomber, Theodore Kaczynski, was considered to have Schizoid Personality Disorder, with bearers of this diagnosis evidencing social detachment and a preference for solitary activities [8].

#### Summary

Absent other psychiatric diagnosis, we hypothesize that Stephen Paddock’s severe Gambling Addiction, (where, statistically, the House always wins), Social Isolation, Sexual Sadism, Antisocial/Psychopathic and Oppositional traits, and reliance on Benzodiazepines/Alcohol, are most economically accounted for by the neuro-genetic rubric, RDS. Reports he planned to escape the carnage suggest some fantasized, (exciting) notoriety. Finally, his self-described awareness that he was “born bad” (alluding to his father’s psychopathy) underscores what might be called, the “ego-alien” (“not my true self”) nature of his symptoms. The missing piece or hole in Paddock could, indeed, be a desensitized brain reward circuit.

## Devin Kelley

News accounts indicate the Sutherland Springs shooter, Devin Kelley, graduated high school. He had been diagnosed with ADHD, with grades ranging from low B to C. During his school years, he was suspended seven times for drug use, insubordination, and making profane gestures or language. (The latter, copropraxia and coprolalia, are motor and vocal tics, respectively, and are consistent with a diagnosis of (possible) Tourette’s Syndrome).

After marrying, he was charged with assaulting his wife and fracturing his toddler step-son’s skull, repeatedly striking, kicking and choking his wife, just months into their marriage. He was observed punching a dog near its head and neck and dragging it away. Reportedly, he bought dogs and other animals and used them for “target practice.” (Reportedly, the Boston Strangler, shot arrows through dogs and cats he trapped as a child and the Columbine shooters mutilated animals for fun).

Such Animal Abuse is consistent with the diagnosis of Conduct Disorder, a precursor to a diagnosis of Anti-Social Personality Disorder (ASPD). In addition, Inter Partner Violence (IPV) is more common in men with ASPD. Moreover, young adult males with ADHD are more likely to engage in partner-directed aggression, with the risk greater among young adult males with *childhood* ADHD. (Approximately 25% of adults with ADHD in childhood meet diagnostic criteria for ASPD disorder.

Kelley’s record of cruelty to animals and intimate partner violence are consistent with diagnoses of Conduct disorder and, probably, ASPD.

### Summary

The co-occurrence of ADHD, possible Tourette’s, Animal Cruelty, Inter Partner Violence, reported Sexual Sadism (rape), early drug use, and “insubordination” (Oppositional Defiant Disorder) can be most readily accounted for by the diagnostic rubric, “Reward Deficiency Syndrome.” This syndrome has well-established, neuro-genetic evidence, indicating that over 110 million Americans have an inherited genetic change, markedly reducing the density of the brain’s main reward receptor, the dopamine D2 receptor.

Nine other reward circuit, genetic polymorphisms (anomalies) have been identified, to date. The inheritance of any of these, in addition to the D2 receptor anomaly, further reduces the Reward Systems’ sensitivity, allowing the emergence of more primitive drives and urges into consciousness. With this stated this hypothesis must be tested by direct analysis of brain tissue from not only these two cases but other mass shooters as well. Our group is actively pursuing thus novel opportunity.

It is our contention that early genetic identification of RDS and its pathological behaviors including hyper –sexuality [10], even in children [11], could be a giant step forward in potentially saving lives.

## Figures and Tables

**Table 1:** Reward Deficiency Syndrome (RDS) behaviors: a function of the reward genes.

Addictive Behaviors		Impulsive Behaviors		Obsessive Compulsive Behaviors	Personality Disorders
Substance Related	Non Substance Related	Spectrum Disorders	Disruptive Impulsive		
Alcohol Cannabis	Thrill-seeking (novelty)	Attention-deficit Hyperactivity	Anti-social	Body Dysmorphic	Paranoid
	Sexual Sadism	Tourette’s and Tic Syndrome	Conduct	Hoarding	Schizoid
Opioids	Sexual Masochism	Autism	Pathological Aggression	Trichotillomania (hair pulling)	Borderline
Sedatives/Hypnotics Stimulants	Hyper-sexuality Pornography Addiction		Oppositional Defiant Exhibitionistic	Excoriation (skin picking)	Schizotypal Histrionic
	Gambling			Non-Suicidal Self Injury	
Tobacco	Internet Gaming				Narcissistic
Glucose					Avoidant
Food			Modified for DSM-V, according to Blum et al. 1996 [9]		Dependent
